# Hospital Admission Trends in Alpha-1-Antitrypsin Deficiency: A Sex-Based Analysis from the Spanish National Discharge Database, 2016–2022

**DOI:** 10.3390/jcm13216564

**Published:** 2024-10-31

**Authors:** Javier de-Miguel-Diez, Ana Lopez-de-Andres, José J. Zamorano-Leon, Valentín Hernández-Barrera, Natividad Cuadrado-Corrales, Ana Jimenez-Sierra, David Carabantes-Alarcon, Rodrigo Jimenez-Garcia

**Affiliations:** 1 Respiratory Care Department, Hospital General Universitario Gregorio Marañón, Universidad Complutense de Madrid, Instituto de Investigación Sanitaria Gregorio Marañón (IiSGM), 28007 Madrid, Spain; javier.miguel@salud.madrid.org; 2 Department of Public Health & Maternal and Child Health, Faculty of Pharmacy, Universidad Complutense de Madrid, 28040 Madrid, Spain; 3 Department of Public Health & Maternal and Child Health, Faculty of Medicine, Universidad Complutense de Madrid, 28040 Madrid, Spain; josejzam@ucm.es (J.J.Z.-L.); mariancu@ucm.es (N.C.-C.); dcaraban@ucm.es (D.C.-A.); rodrijim@ucm.es (R.J.-G.); 4 Preventive Medicine and Public Health Teaching and Research Unit, Health Sciences Faculty, Rey Juan Carlos University, 28922 Madrid, Spain; valentin.hernandez@urjc.es; 5 Faculty of Medicine, Universidad San Pablo Ceu, 28668 Madrid, Spain; a.jimenez100@usp.ceu.es

**Keywords:** alpha-1 antitrypsin deficiency, hospital admissions, comorbidities, in-hospital mortality, sex-differences, trends

## Abstract

**Objectives**: To analyze the number and clinical characteristics of hospital admissions in Spain between 2016 and 2022 in which alpha-1-antitrypsin deficiency (AATD) was coded; to describe and analyze differences in these parameters between men and women; and to identify variables associated with a worse prognosis. **Methods**: We used a nationwide discharge database to select all admissions featuring an AATD diagnostic code (ICD-10 code E88.01) in any position. **Results**: We found 5142 hospital admissions with a diagnosis of AATD and detected a significant increase in their number from 2016 to 2022 (*p* = 0.034 for trend). Males accounted for 58.21% of the hospitalizations and had a higher Charlson Comorbidity Index than women (1.86 vs. 1.33; *p* < 0.001), were hospitalized more frequently (21.18% of men were hospitalized more than once vs. 17.76% of women, *p* < 0.001), and had a higher probability of severe disease (OR 1.39; 95%CI 1.10–1.75). Crude in-hospital mortality (IHM) was 6.85% in men and 4.8% in women (*p* = 0.007). The variables associated with IHM in both sexes were older age, more hospital admissions, and liver disease or lung cancer. Invasive and non-invasive mechanical ventilation and admission to the ICU were also associated with IHM in men and women. Multivariable adjustment revealed no association between sex and IHM. **Conclusions**: The number of hospitalizations for AATD increased in Spain from 2016 to 2022. Men represented almost 60% of hospitalizations, were admitted more frequently and with more comorbidities, and had a higher probability of severe disease than women. There was no association between sex and IHM.

## 1. Introduction

Alpha-1 antitrypsin (A1AT) is a 52-kDa, acute phase glycoprotein encoded by the protease inhibitor (PI) locus, located on the long arm of chromosome 14 (14q31–32.3) [1]. Alpha-1-antitrypsin deficiency (AATD) is an inherited, autosomal co-dominant genetic disorder that is characterized by low serum levels of alpha-1-antitrypsin, the most abundant protease inhibitor in serum. Alpha-1-antitrypsin is produced and secreted in the liver, but has an important physiological function in the lungs, protecting the alveoli from damage. Therefore, AATD is associated with pulmonary and liver diseases [2,3,4].

Chronic obstructive pulmonary disease (COPD) and emphysema are the most common disorders affecting the respiratory system of patients with AATD, although other conditions, such as asthma or bronchiectasis, may also be observed [5,6]. Environmental factors, particularly tobacco smoke, significantly increase the risk of developing COPD. Consequently, the onset of respiratory disease in smokers appears in the third or fourth decade of life, while in non-smokers, it may not appear until the fifth or sixth decade. Some non-smokers may even have a normal life span without developing COPD or other disorders associated with AATD [7].

Many of the issues related to sex and gender in COPD are likely to be relevant to AATD. For example, occupational exposures and certain risk behaviors, such as tobacco and alcohol use, which still show gender differences, have a significant impact on the development of lung and liver diseases in AATD patients [8].

AATD is a largely unrecognised disease [9]. Clinical practice guidelines, both national [10] and international [11], recommend assessment of alpha-1-antitrypsin, at least once during the lifetime of patients with COPD, emphysema, or other respiratory conditions presenting with not completely reversible airflow obstruction and liver disease of unknown etiology. Nevertheless, the number of patients diagnosed with AATD is much smaller than expected, and diagnosis remains unconfirmed in most cases until late in the course of the lung or liver disease, after many visits to various physicians [12].

The prevalence of AATD has been reported to vary appreciably throughout the world, probably because of variations in national prevalence, in screening activities, in data sources, in case definitions, and in methods of analysis (especially the duration of the study period). If data from different studies are extrapolated, the estimated prevalence of AATD ranges from 1:2500 to 1:5000 [6,13,14].

The lack of large patient cohorts hampers our understanding of the clinical features and natural history of rare diseases. Analyses of large healthcare databases could provide important information in this regard. In the present study, we used the Spanish Hospital Discharge Database (SHDD) to analyze the number and clinical characteristics of hospital admissions occurring between 2016 and 2022 in Spain in which AATD was coded. Finally, variables associated with a worse prognosis in men and women with AATD were identified.

## 2. Materials and Methods

### 2.1. Design and Data Source

A descriptive, retrospective, observational study was performed. Data were extracted from the SHDD. The recruitment period spanned from 1 January 2016 to 31 December 2022. The SHDD collects basic demographic data, up to 20 diagnoses and procedures, admission to the intensive care unit (ICU), admission and discharge dates, and the outcome of hospitalization (death, discharge, or transfer to another health center). Detailed information on the SHDD can be found elsewhere [15].

The diagnoses included in the database are those present upon admission or made during hospitalization. The procedures recorded are both diagnostic and therapeutic. The SHDD used the International Classification of Diseases, 10th Revision (ICD-10).

### 2.2. Participants

All admissions featuring an AATD diagnostic code (ICD-10 code E88.01) in any diagnostic position were selected. Admissions lacking age, sex, admission or discharge dates, or reasons for discharge were excluded. For the cohort study, patients who were admitted at least once with a diagnosis of AATD over the seven-year study period were identified. As the database is anonymized, an algorithm including date of birth, sex, and place of residence was used to determine whether the same patient had been admitted more than once.

### 2.3. Variables

For the descriptive study, the outcome measures of our research were the number and characteristics of AATD admissions in Spain from 2016 to 2022. In the cohort study, in-hospital mortality (IHM) was defined as the main outcome measure.

Covariates included sex, age, number of admissions during the study period, Charlson Comorbidity Index (CCI), specific comorbidities present at the time of admission or that developed during hospitalization (shown in Table S1 along with their ICD-10 codes), use of invasive and non-invasive mechanical ventilation, length of hospital stay, admission to the ICU, and severity. The number of admissions was categorized into one, two, and three or more. The codes used for calculating the Charlson Comorbidity Index have been published elsewhere [16,17] and were analyzed by categorizing them into three groups based on the number of conditions (none, one, two or more).

The condition of patients who required admission to the ICU and/or died in the hospital was considered severe.

In the cohort study, all the above-mentioned covariates were analyzed as exposure variables, with the population stratified by sex. To evaluate the effect of sex, the analysis was repeated with the entire study population.

### 2.4. Statistical Analysis

We recorded and compared the total number of hospital admissions with AATD by year and according to the study variables. Differences between men and women hospitalized with AATD were also analyzed.

Quantitative variables are expressed as the mean with its standard deviation or the median with the interquartile range (IQR). Qualitative variables are expressed as absolute frequencies and percentages.

The methods used to observe potential changes in the distribution of the variables from 2016 to 2022 included the linear regression *t* test or Jonckheere–Terpstra test for quantitative variables, and the Cochran–Mantel–Haenszel statistic or Cochran–Armitage test for qualitative variables.

If quantitative variables conformed to normality according to the Kolmogorov–Smirnov test, they were compared using the t test; otherwise, the Mann–Whitney test was applied. The Fisher exact test was used to compare proportions.

In the cohort study, variables associated with IHM were identified using logistic regression models. Three models were generated, one for each sex and another for the entire population. The measure of association obtained was the odds ratio (OR) with its 95% confidence interval (CI).

The analysis was performed using Stata version 14 (Stata, College Station, TX, USA). Statistical significance was set at *p* < 0.05 (2-tailed).

### 2.5. Sensitivity Analysis

The multivariable analyses were repeated using severity as the dependent variable to confirm the variables associated with a worse prognosis (admission to the ICU or death).

### 2.6. Ethics Statement

SHDD data are collected and purged by the Spanish Ministry of Health. The Ministry assesses the relevance and compliance with ethical requirements of requests for the database made by researchers online [18]. If the Ministry of Health considers the proposal appropriate, it supplies the requested database, which is fully anonymized. According to Spanish legislation, as the SHDD is an administrative database, patients are not required to sign an informed consent document before inclusion.

## 3. Results

### 3.1. Course and Characteristics of Hospitalizations with an AATD Code

Table 1 shows the number and characteristics of hospitalizations of patients with an AATD code in Spain over the seven years studied. A total of 5142 hospital admissions with an AATD diagnosis were recorded. The lowest number of admissions was in 2016, with 576, increasing to 967 in 2022. The increase was steady except for 2020 (682), when a significant decrease was observed compared to the previous year.

The mean age of admissions was approximately 58 years, increasing from 56.34 years in 2016 to 59.31 in 2022 (*p* < 0.001). Males accounted for 58.21% of hospitalizations, although the proportion of women increased significantly over time.

The most frequently coded pathology in AATD admissions was COPD, which was diagnosed in 58.03% of hospitalizations; this prevalence remained stable over time. Emphysema was the second most common diagnosis (25.75%), although this diagnosis decreased from 29.51% in 2016 to 22.75% in 2022 (*p* < 0.001). The frequency of bronchiectasis, on the other hand, increased from 6.6% to 11.27% (*p* = 0.035) over the study period. The prevalence of liver disease remained stable, at close to 16%.

Since its emergence in the year 2020, COVID-19 was coded in almost 10% of admissions, with no changes over time. The prevalence of pneumonia also remained stable, fluctuating between 6% and 9%.

The percentage of hospitalizations requiring admission to the ICU or resulting in death were 8.05% and 4.55%, respectively, with no significant temporal trends observed in either variable. In 11.4% of cases, the presenting complaint was classified as severe.

### 3.2. Characteristics of and Differences Between Men and Women Admitted with AATD

A total of 3922 individual patients were hospitalized with AATD in Spain between 2016 and 2022 (2233 men [56.93%] and 1689 women [43.07%]). The comparison by sex is presented in Table 2. Mean age and CCI values were higher in men than in women (58.26 years vs. 55.92 years [*p* < 0.001] and 1.86 vs. 1.33 [*p* < 0.001]). Men were hospitalized more frequently than women, with the percentage hospitalized more than once being 21.18% for men and 17.76% for women (*p* < 0.001). A comparison of the conditions diagnosed reveals higher prevalence values in men for COPD, emphysema, obstructive sleep apnea, liver disease, diabetes, hypertension, chronic kidney disease, and lung cancer. Women more frequently presented with bronchiectasis, asthma, depression, osteoporosis, gastro-oesophageal reflux disease, and obesity.

A comparison of variables for outcome of hospital stay showed that men required more invasive mechanical ventilation (2.64% vs. 1.54%; *p* = 0.019) and admission to the ICU (10.48% vs. 6.22%; *p* = 0.02). The crude IHM was 6.85% in men and 4.8% in women (*p* = 0.007), with the percentage of cases classified as severe being 15.49% and 9.77%, respectively (*p* < 0.001). The length of hospital stay was also significantly longer for men (median 6 days vs. 5 days; *p* < 0.001).

### 3.3. Variables Associated with IHM in Men and Women with AATD: Bivariate Analysis

Table 3 shows the distribution of study variables in men admitted with AATD based on their survival at hospital admission. As observed, the mean age of the deceased was 13 years higher than among survivors (70.98 years vs. 57.33 years; *p* < 0.001). Men with three or more admissions had an IHM of 14.02% compared to 5.57% for those admitted only once (*p* < 0.001). The mean CCI value was more than double among the deceased (3.65 vs. 1.72; *p* < 0.001). The analysis of comorbidities showed that having a diagnosis of congestive heart failure, myocardial infarction, chronic kidney disease, diabetes, liver disease, lung cancer, and COPD was significantly associated with IHM.

Among men diagnosed with COVID-19, the IHM was 12.6%, with this proportion being 12.3% among those who developed pneumonia.

The need for invasive or non-invasive mechanical ventilation or admission to the ICU was also associated with a higher IHM.

Variables associated with IHM in women hospitalized with an AATD code are summarized in Table 4. Similar to men, the mean age and CCI were significantly higher among deceased women (70.94 years vs. 55.16 years [*p* < 0.001] and 2.84 vs. 1.26 [*p* < 0.001]). The IHM among those admitted three or more times rose to 11.11% compared to only 3.6% in those with a single admission (*p* < 0.001).

The concomitant conditions associated with IHM were congestive heart failure, chronic kidney disease, liver disease, and pneumonia. Invasive and non-invasive mechanical ventilation and admission to the ICU were also associated with higher IHM.

### 3.4. Variables Associated with IHM After Multivariable Analysis

Multivariable adjustment with logistic regression revealed the variables associated with IHM in men, women, and the entire population (Table 5) to be older age and more hospital admissions. Among the concomitant conditions, the only predictors of mortality for which differences reached statistical significance in both men and women were liver disease and lung cancer.

The variables associated with higher IHM among men were congestive heart failure, COVID-19 (OR 2.66; 95%CI 1.33–5.34), and pneumonia (2.14; 95%CI 1.22–3.74).

Both invasive and non-invasive mechanical ventilation and admission to the ICU were associated with IHM in men, women, and the entire study sample.

Multivariable adjustment revealed no association between sex and IHM.

### 3.5. Sensitivity Analysis Results

By analyzing variables associated with presenting a case classified as severe in men, women, and both sexes using multivariable regression, we obtained the results shown in Table S2. The variables associated with severity were very similar to those identified for IHM, such as older age, more admissions, congestive heart failure, COVID-19, and pneumonia.

Being male was associated with a higher probability of severe disease (OR 1.39; 95%CI 1.10–1.75).

## 4. Discussion

Our study revealed an increase in the number of hospitalizations for AATD in Spain from 2016 to 2022. The increase was constant, except in the year 2020, in which there was a substantial decrease in relation to the previous year, as reported in hospital admissions for acute exacerbations of COPD during the pandemic [19], suggesting that these patients may have avoided seeking medical attention for fear of COVID-19 infection. Similar trends have been reported elsewhere. Acquavella et al. [6] found an increase in the prevalence of AATD in Denmark from 2000 to 2018, attributing it to increased medical awareness. As in our case, prevalence was higher in men than in women, probably due to a higher likelihood of diagnosis in men, as AATD is not sex-related. Higher smoking rates in men probably accelerate the manifestations of lung and other diseases [6].

The most common diseases related to AATD in our study were respiratory disorders, mainly COPD and emphysema. This finding is in line with the known predominance of pulmonary manifestations throughout the lifetime of patients with AATD [6,20]. However, as in our study, several authors have reported other lung phenotypes, such as asthma and bronchiectasis [21,22,23]. Notably, smoking can significantly exacerbate respiratory diseases and is the main risk factor for rapidly progressive COPD in people with AATD [24].

In addition to lung complications, AATD is associated with a variety of disorders, including liver disease, as detected in the current study. Our data also revealed that individuals with AATD had a higher prevalence of arterial hypertension, diabetes, and chronic kidney disease, although the frequency of myocardial infarction was lower. Similarly, Zöller et al. [25] found that patients with AATD-related COPD had a lower risk of cardiovascular disease than COPD patients without AATD.

The comorbidities of AATD reflect key risk factors that could predispose patients to severe COVID-19 [26]. Nevertheless, recent studies suggest that the risk for infection, severity, or death from COVID-19 is no greater in patients with AATD than in the general population [27]. In our study, COVID-19 was diagnosed, since it first appeared, in approximately 1 in 10 hospital admissions. The prevalence of pneumonia also remained stable over time.

Patients with AATD may present with a broad spectrum of organ-specific complications, thus necessitating admission to the ICU [28]. Our data revealed that 8.05% and 4.55%, respectively, of patients hospitalized required admission to the ICU or died in-hospital, with no significant temporal variations being observed for either of the two variables. The condition leading to admission was classified as severe (transfer to ICU and/or IHM). In 11.4% of cases, this percentage also remained stable over time.

Data on the impact of sex on the clinical presentation of patients with AATD, disease course, and other clinical outcomes are limited. In our study, men accounted for almost 60% of hospitalizations and were also admitted more frequently and with more comorbidities. In the same line, Acquavella et al. [6] found a diagnosed AATD incidence 21% higher for males than for females and attributed it to higher smoking rates in men, as we have previously indicated.

Men had a higher prevalence of COPD and emphysema, obstructive sleep apnea, liver disease, diabetes, hypertension, chronic kidney disease, and lung cancer. Women, on the other hand, more frequently presented bronchiectasis, asthma, depression, osteoporosis, gastro-oesophageal reflux disease, and obesity. In COPD, bronchitis has been reported to be more frequent in females, whereas emphysema is more common in males [29]. However, in an analysis from the German registry, Fähndrich et al. [30] did not find a clear sex-specific predominant phenotype in individuals with AATD.

Few data on mortality have been reported in AATD. Previous studies report that severe AATD reduces life expectancy and that respiratory failure and liver disease are the leading causes of death [31,32,33]. More recent studies have also demonstrated increased mortality due to heart failure and decreased mortality due to ischaemic heart disease in patients with severe AATD than in the general population [34]. In our study, the predictors of IHM in both sexes were older age, more frequent admission to hospital, liver disease, and lung cancer. Among men, congestive heart failure and pneumonia were associated with higher IHM, as was COVID-19 infection. However, other authors have not been able to detect a worse prognosis of COVID-19 in patients with AATD [35,36]. The use of invasive and non-invasive mechanical ventilation and admission to the ICU were associated with IHM in men, women, and in the entire sample studied. Multivariable adjustment revealed no association between sex and IHM, as reported by other authors [34]. Based on severity as the outcome measure, the sensitivity analysis confirmed the variables identified for IHM, and found that male sex was associated with greater severity.

The major strengths of this study are the large cohort of patients with AATD provided by the SHDD and the long study period. However, our analysis is limited by its retrospective design, the dependence on accurate ICD-10 coding, and the data available. Consequently, we do not have information about AATD genotype, disease severity, lung function, laboratory tests, smoking, or augmentation therapy. However, given that the SHDD integrates data from both public and private hospitals, regardless of their healthcare purpose, the resulting large study sample enables us to present robust findings [37].

## 5. Conclusions

In conclusion, the number of hospitalizations for AATD increased in Spain from 2016 to 2022. Men accounted for almost 60% of hospitalizations and were admitted more frequently and with more comorbidities. The predictors of IHM in both sexes were older age, more frequent hospital admissions, liver disease, and lung cancer. Mechanical ventilation and admission to the ICU were also associated with IHM in both men and women. There was no association between sex and IHM.

## Figures and Tables

**Table 1:** Distribution of hospital admissions with Alpha-1-Antitrypsin deficiency in Spain by year (2016–2022) and according to main study variables.

		Year								
		2016	2017	2018	2019	2020	2021	2022	Total	*p* for Trend
Total (n)		576	658	741	770	682	748	967	5142	
Age, mean (SD)		56.34 (20.07)	56.71 (20.76)	58.65 (19.6)	57.93 (20.69)	58.82 (19.54)	58.71 (18.99)	59.31 (18.77)	58.19 (19.74)	0.034
Age groups, n (%)	<30 years	58 (10.07)	71 (10.79)	62 (8.37)	76 (9.87)	61 (8.94)	63 (8.42)	76 (7.86)	467(9.08)	0.133
	30–49 years	89 (15.45)	114 (17.33)	113 (15.25)	127 (16.49)	91 (13.34)	135 (18.05)	156 (16.13)	825 (16.04)	
	50–69 years	272 (47.22)	266 (40.43)	322 (43.45)	317 (41.17)	322 (47.21)	305 (40.78)	415 (42.92)	2219 (43.15)	
	≥70 years	157 (27.26)	207 (31.46)	244 (32.93)	250 (32.47)	208 (30.5)	245 (32.75)	320 (33.09)	1631 (31.72)	
Sex, n (%)	Male	364 (63.19)	391 (59.42)	438 (59.11)	427 (55.45)	380 (55.72)	450 (60.16)	543 (56.15)	2993 (58.21)	0.035
	Female	212 (36.81)	267 (40.58)	303 (40.89)	343 (44.55)	302 (44.28)	298 (39.84)	424 (43.85)	2149 (41.79)	
Hypertension, n (%)		151 (26.22)	147 (22.34)	171 (23.08)	205 (26.62)	176 (25.81)	195 (26.07)	269 (27.82)	1314 (25.55)	0.158
Congestive heart failure, n (%)		29 (5.03)	42 (6.38)	54 (7.29)	70 (9.09)	53 (7.77)	68 (9.09)	101 (10.44)	417 (8.11)	0.003
Myocardial infarction, n (%)		13 (2.26)	9 (1.37)	10 (1.35)	15 (1.95)	11 (1.61)	26 (3.48)	41 (4.24)	125 (2.43)	<0.001
Chronic renal disease, n (%)		35 (6.08)	39 (5.93)	55 (7.42)	54 (7.01)	37 (5.43)	76 (10.16)	76 (7.86)	372 (7.23)	0.012
Depression, n (%)		15 (2.6)	17 (2.58)	20 (2.7)	19 (2.47)	20 (2.93)	19 (2.54)	37 (3.83)	147 (2.86)	0.629
Diabetes, n (%)		64 (11.11)	101 (15.35)	97 (13.09)	113 (14.68)	125 (18.33)	118 (15.78)	124 (12.82)	742 (14.43)	0.006
Osteoporosis, n (%)		20 (3.47)	24 (3.65)	27 (3.64)	34 (4.42)	20 (2.93)	32 (4.28)	50 (5.17)	207 (4.03)	0.340
GORD, n (%)		22 (3.82)	7 (1.06)	17 (2.29)	34 (4.42)	34 (4.99)	34 (4.55)	38 (3.93)	186 (3.62)	0.001
Liver disease, n (%)		96 (16.67)	101 (15.35)	108 (14.57)	110 (14.29)	127 (18.62)	114 (15.24)	158 (16.34)	814 (15.83)	0.317
Lung cancer, n (%)		5 (0.87)	10 (1.52)	15 (2.02)	13 (1.69)	14 (2.05)	14 (1.87)	11 (1.14)	82 (1.59)	0.489
COPD, n (%)		346 (60.07)	384 (58.36)	457 (61.67)	451 (58.57)	379 (55.57)	413 (55.21)	554 (57.29)	2984 (58.03)	0.148
Asthma, n (%)		41 (7.12)	71 (10.79)	75 (10.12)	92 (11.95)	79 (11.58)	85 (11.36)	112 (11.58)	555 (10.79)	0.094
Emphysema, n (%)		170 (29.51)	196 (29.79)	222 (29.96)	177 (22.99)	161 (23.61)	178 (23.8)	220 (22.75)	1324 (25.75)	<0.001
Bronchiectasis, n (%)		38 (6.6)	71 (10.79)	93 (12.55)	84 (10.91)	79 (11.58)	82 (10.96)	109 (11.27)	556 (10.81)	0.035
COVID-19, n (%)		NA	NA	NA	NA	69 (10.12)	71 (9.49)	103 (10.65)	243 (4.76)	0.0236
Pneumonia, n (%)		41 (7.12)	57 (8.66)	67 (9.04)	66 (8.57)	55 (8.06)	45 (6.02)	70 (7.24)	401 (7.8)	0.313
Obesity, n (%)		33 (5.73)	48 (7.29)	53 (7.15)	76 (9.87)	76 (11.14)	76 (10.16)	113 (11.69)	475 (9.24)	<0.001
OSA, n (%)		26 (4.51)	43 (6.53)	49 (6.61)	69 (8.96)	62 (9.09)	61 (8.16)	96 (9.93)	406 (7.9)	0.002
Invasive mechanical ventilation, n (%)		11 (1.91)	10 (1.52)	12 (1.62)	14 (1.82)	18 (2.64)	18 (2.41)	14 (1.45)	97 (1.89)	0.548
Non-invasive mechanical ventilation, n (%)		9 (1.56)	15 (2.28)	14 (1.89)	18 (2.34)	22 (3.23)	24 (3.21)	26 (2.69)	128 (2.49)	0.359
LOHS, median (IQR)		6 (7)	6 (7)	6 (8)	5 (7)	6 (8)	6 (8)	6 (7)	6 (7)	0.526
Admission to ICU, n (%)		43 (7.47)	46 (6.99)	60 (8.1)	71 (9.22)	52 (7.62)	64 (8.56)	78 (8.07)	414 (8.05)	0.794
In-hospital mortality, n (%)		22 (3.82)	26 (3.95)	31 (4.18)	29 (3.77)	30 (4.4)	47 (6.28)	49 (5.07)	234 (4.55)	0.212
Severity, n (%)		59 (10.24)	69 (10.49)	82 (11.07)	87 (11.3)	76 (11.14)	100 (13.37)	113 (11.69)	586 (11.4)	0.624

GORD: gastro-oesophageal reflux disease. COPD: chronic obstructive pulmonary disease. OSA: obstructive sleep apnea. LOHS: length of hospital stay. IQR: interquartile range. ICU: Intensive care unit. NA: not available.

**Table 2:** Characteristics of patients hospitalized with Alpha-1-Antitrypsin deficiency in Spain (2016–2022) according to sex.

		TOTAL	MEN	WOMEN	*p*
Number of individual patients, No. (%)		3922	2233 (56.93)	1689 (43.07)	<0.001
Age, mean (SD)		57.25 (20.11)	58.26 (19.27)	55.92 (21.1)	<0.001
Age groups, No. (%)	<30 years	393 (10.02)	202 (9.05)	191(11.31)	<0.001
	30–49 years	685 (17.47)	328 (14.69)	357 (21.14)	
	50–69 years	1673 (42.66)	1026 (45.95)	647 (38.31)	
	≥70 years	1171 (29.86)	677 (30.32)	494(29.25)	
Number of hospitalizations, n (%)	One	3149 (80.29)	1760 (78.82)	1389 (82.24)	0.012
	Two	519 (13.23)	309 (13.84)	210 (12.43)	
	Three or more	254 (6.48)	164 (7.34)	90 (5.33)	
Charlson Comorbidity Index, n (%)	None	892 (22.74)	395 (17.69)	497 (29.43)	<0.001
	One	1580 (40.29)	877 (39.27)	703 (41.62)	
	Two or more	1450 (36.97)	961 (43.04)	489 (28.95)	
Charlson Comorbidity Index, mean (SD)		1.63 (1.69)	1.86 (1.79)	1.33 (1.49)	<0.001
Hypertension, n (%)		984 (25.09)	602 (26.96)	382 (22.62)	0.002
Congestive heart failure, n (%)		291 (7.42)	159 (7.12)	132 (7.82)	0.411
Myocardial infarction, n (%)		95 (2.42)	63 (2.82)	32 (1.89)	0.062
Chronic renal disease, n (%)		257 (6.55)	166 (7.43)	91 (5.39)	0.010
Depression, n (%)		121 (3.09)	44 (1.97)	77 (4.56)	<0.001
Diabetes, n (%)		549 (14)	364 (16.3)	185 (10.95)	<0.001
Osteoporosis, n (%)		162 (4.13)	41 (1.84)	121 (7.16)	<0.001
GORD, n (%)		127 (3.24)	61 (2.73)	66 (3.91)	0.039
Liver disease, n (%)		570 (14.53)	395 (17.69)	175 (10.36)	<0.001
Lung cancer, n (%)		65 (1.66)	51 (2.28)	14 (0.83)	<0.001
COPD, n (%)		2209 (56.32)	1326 (59.38)	883 (52.28)	<0.001
Asthma, n (%)		459 (11.7)	166 (7.43)	293 (17.35)	<0.001
Emphysema, n (%)		949 (24.2)	640 (28.66)	309 (18.29)	<0.001
Bronchiectasis, n (%)		407 (10.38)	178 (7.97)	229 (13.56)	<0.001
COVID-19, n (%)		211 (5.38)	127 (5.69)	84 (4.97)	0.326
Pneumonia, n (%)		302 (7.7)	187 (8.37)	115 (6.81)	0.069
Obesity, n (%)		356 (9.08)	184 (8.24)	172 (10.18)	0.036
OSA, n (%)		306 (7.8)	218 (9.76)	88 (5.21)	<0.001
Invasive mechanical ventilation, n (%)		85 (2.17)	59 (2.64)	26 (1.54)	0.019
Non-invasive mechanical ventilation, n (%)		96 (2.45)	59 (2.64)	37 (2.19)	0.365
LOHS, median (IQR)		6 (7)	6 (7)	5 (7)	<0.001
Admission to ICU, n (%)		339 (8.64)	234 (10.48)	105 (6.22)	0.020
In-hospital mortality, n (%)		234 (5.97)	153 (6.85)	81 (4.8)	0.007
Severity, n (%)		511 (13.03)	346 (15.49)	165 (9.77)	<0.001

GORD: gastro-oesophageal reflux disease. COPD: chronic obstructive pulmonary disease. OSA: obstructive sleep apnea. LOHS: length of hospital stay. IQR: interquartile range. ICU: intensive care unit.

**Table 3:** Characteristics of male patients hospitalized with Alpha-1-Antitrypsin deficiency in Spain (2016–2022) according to survival to hospital admission.

		SURVIVED	DIED	*p*
Number of individual patients, (%)		2080 (93.15)	153 (6.85)	<0.001
Age, mean (SD)		57.33 (19.39)	70.98 (11.52)	<0.001
Age groups, No. (%)	<30 years	202 (100)	0 (0)	<0.001
	30–49 years	318 (96.95)	10 (3.05)	
	50–69 years	974 (94.93)	52 (5.07)	
	≥70 years	586 (86.56)	91 (13.44)	
Number of hospitalizations, n (%)	One	1662 (94.43)	98 (5.57)	<0.001
	Two	277 (89.64)	32 (10.36)	
	Three or more	141 (85.98)	23 (14.02)	
Charlson Comorbidity Index, n (%)	None	390 (98.73)	5 (1.27)	<0.001
	One	846 (96.47)	31 (3.53)	
	Two or more	844 (87.83)	117 (12.17)	
Charlson Comorbidity Index, mean (SD)		1.72 (1.66)	3.65 (2.47)	<0.001
Hypertension, n (%)		562 (93.36)	40 (6.64)	0.814
Congestive heart failure, n (%)		126 (79.25)	33 (20.75)	<0.001
Myocardial infarction, n (%)		52 (82.54)	11 (17.46)	0.001
Chronic renal disease, n (%)		143 (86.14)	23 (13.86)	0.000
Depression, n (%)		38 (86.36)	6 (13.64)	0.072
Diabetes, n (%)		329 (90.38)	35 (9.62)	0.023
Osteoporosis, n (%)		37 (90.24)	4 (9.76)	0.457
GORD, n (%)		60 (98.36)	1 (1.64)	0.102
Liver disease, n (%)		344 (87.09)	51 (12.91)	<0.001
Lung cancer, n (%)		44 (86.27)	7 (13.73)	0.049
COPD, n (%)		1214 (91.55)	112 (8.45)	<0.001
Asthma, n (%)		157 (94.58)	9 (5.42)	0.448
Emphysema, n (%)		586 (91.56)	54 (8.44)	0.060
Bronchiectasis, n (%)		167 (93.82)	11 (6.18)	0.711
COVID-19, n (%)		111 (87.4)	16 (12.6)	0.008
Pneumonia, n (%)		164 (87.7)	23 (12.3)	0.002
Obesity, n (%)		170 (92.39)	14 (7.61)	0.671
OSA, n (%)		205 (94.04)	13 (5.96)	0.585
Invasive mechanical ventilation, n (%)		34 (57.63)	25 (42.37)	<0.001
Non-invasive mechanical ventilation, n (%)		47 (79.66)	12 (20.34)	<0.001
LOHS, median (IQR)		6 (7)	9 (15)	<0.001
Admission to ICU, n (%)		193 (82.48)	41 (17.52)	<0.001

*p* differences for those who survived vs. those that died in the hospital. GORD: gastro-oesophageal reflux disease. COPD: chronic obstructive pulmonary disease. OSA: obstructive sleep apnea. LOHS: length of hospital stay. IQR: interquartile range. ICU: intensive care unit.

**Table 4:** Characteristics of female patients hospitalized with Alpha-1-Antitrypsin deficiency in Spain (2016–2022) according to survival to hospital admission.

		SURVIVED	DIED	*p*
Number of individual patients, (%)		1608 (95.2)	81 (4.8)	<0.001
Age, mean (SD)		55.16 (20.98)	70.94 (17.69)	<0.001
Age groups, No. (%)	<30 years	188 (98.43)	3 (1.57)	<0.001
	30–49 years	354 (99.16)	3 (0.84)	
	50–69 years	624 (96.45)	23 (3.55)	
	≥70 years	442 (89.47)	52 (10.53)	
Number of hospitalizations, n (%)	One	1339 (96.4)	50 (3.6)	<0.001
	Two	189 (90)	21 (10)	
	Three or more	80 (88.89)	10 (11.11)	
Charlson Comorbidity Index, n (%)	None	493 (99.2)	4 (0.8)	<0.001
	One	677 (96.3)	26 (3.7)	
	Two or more	438 (89.57)	51 (10.43)	
Charlson Comorbidity Index, mean (SD)		1.26 (1.4)	2.84 (2.29)	<0.001
Hypertension, n (%)		356 (93.19)	26 (6.81)	0.037
Congestive heart failure, n (%)		115 (87.12)	17 (12.88)	0.000
Myocardial infarction, n (%)		30 (93.75)	2 (6.25)	0.697
Chronic renal disease, n (%)		77 (84.62)	14 (15.38)	0.000
Depression, n (%)		74 (96.1)	3 (3.9)	0.705
Diabetes, n (%)		171 (92.43)	14 (7.57)	0.062
Osteoporosis, n (%)		115 (95.04)	6 (4.96)	0.931
GORD, n (%)		63 (95.45)	3 (4.55)	0.923
Liver disease, n (%)		152 (86.86)	23 (13.14)	0.000
Lung cancer, n (%)		12 (85.71)	2 (14.29)	0.095
COPD, n (%)		838 (94.9)	45 (5.1)	0.545
Asthma, n (%)		279 (95.22)	14 (4.78)	0.988
Emphysema, n (%)		291 (94.17)	18 (5.83)	0.349
Bronchiectasis, n (%)		215 (93.89)	14 (6.11)	0.315
COVID-19, n (%)		80 (95.24)	4 (4.76)	0.988
Pneumonia, n (%)		105 (91.3)	10 (8.7)	0.043
Obesity, n (%)		160 (93.02)	12 (6.98)	0.158
OSA, n (%)		83 (94.32)	5 (5.68)	0.689
Invasive mechanical ventilation, n (%)		11 (42.31)	15 (57.69)	<0.001
Non-invasive mechanical ventilation, n (%)		29 (78.38)	8 (21.62)	<0.001
LOHS, median (IQR)		5 (6)	9 (18)	<0.001
Admission to ICU, n (%)		84 (80)	21 (20)	<0.001

*p* differences for those who survived vs. those that died in the hospital. GORD: gastro-oesophageal reflux disease. COPD: chronic obstructive pulmonary disease. OSA: obstructive sleep apnea. LOHS: length of hospital stay. IQR: interquartile range. ICU: intensive care unit.

**Table 5:** Multivariable logistic regression to identify factors associated with in-hospital mortality in men and women hospitalized with a code for Alpha-1-Antitrypsin deficiency in Spain from 2016 to 2022.

		MEN	WOMEN	BOTH
		OR (95%CI)	OR (95%CI)	OR (95%CI)
Age groups, No. (%)	<30 years	Reference	Reference	Reference
	30–49 years	Reference	0.3 (0.04–2.05)	1.56 (0.4–6.07)
	50–69 years	1.41 (0.66–3)	1.22 (0.29–5.21)	1.97 (0.84–3.53)
	>70 years	4.29 (2.01–9.16)	4.4 (1.02–19.07)	4.35 (1.54–8.97)
Number of hospitalizations	One	Reference	Reference	Reference
	Two	1.77 (1.1–2.85)	2.8 (1.49–5.25)	1.98 (1.37–2.86)
	Three or more	2.34 (1.36–4.06)	2.92 (1.25–6.83)	2.34 (1.49–3.68)
Congestive heart failure	Yes	2.18 (1.31–3.63)	-	1.86 (1.23–2.8)
Liver disease	Yes	1.96 (1.22–3.15)	2.02 (1–4.08)	2.01 (1.4–3.04)
Lung cancer	Yes	2 (1.01–5.01)	6.36 (1.12–36.19)	2.37 (1.07–5.25)
COVID-19	Yes	2.66 (1.33–5.34)	-	1.78 (1.02–3.2)
Pneumonia	Yes	2.14 (1.22–3.74)	-	1.86 (1.18–2.92)
Invasive mechanical ventilation	Yes	9.93 (4.53–21.78)	13.95 (6.94–32.68)	10.79 (5.72–20.37)
Non-invasive mechanical ventilation	Yes	3.27 (1.45–7.37)	4.18 (1.4–12.47)	3.52 (1.72–6.03)
Admission to ICU	Yes	2.36 (1.33–4.17)	3.2 (1.3–7.86)	2.66 (1.54–3.93)
SEX	Men	NA	NA	1.07 (0.77–1.5)

OR: odds Ratio. CI: confidence interval. ICU: intensive care unit. NA: not available.

## Data Availability

According to the contract signed with the Spanish Ministry of Health and Social Services, which provided access to the databases from the Spanish National Hospital Database, we cannot share the databases with any other investigator, and we have to destroy the databases once the investigation has concluded. Consequently, we cannot upload the databases to any public repository. However, any investigator can apply for access to the databases by filling out the questionnaire available at https://www.sanidad.gob.es/estadEstudios/estadisticas/estadisticas/estMinisterio/SolicitudCMBD.htm (accessed on 20 June 2024). All other relevant data are included in this paper.

## Supplementary Materials

The following supporting information can be downloaded at: https://www.mdpi.com/article/10.3390/jcm13216564/s1, Table S1: ICD10 codes used in this investigation; Table S2: Multivariable logistic regression to identify factors associated with severity in men and women hospitalized with a code for Alpha-1-Antitrypsin deficiency in Spain from 2016 to 2022.
