# Supplementary material for: Hospital Admission Trends in Alpha-1-Antitrypsin Deficiency: A Sex-Based Analysis from the Spanish National Discharge Database, 2016–2022
Source: J Clin Med. 2024 Oct 31;13(21):6564. doi: 10.3390/jcm13216564 (PMC11547007; doi:10.3390/jcm13216564)
Supplement: Supplementary file 1 [file jcm-13-06564-s001.zip › jcm-3247117-supplementary.pdf]

Table S1. ICD10 codes used in this investigation.

Diagnosis or procedure	IC10 codes
Alpha 1-Antitrypsin deficiency	E88.01
Arterial HTN	I10
Congestive Heart Failure	I50
Myocardial infarction	I21, I22
Chronic Renal disease	N18
Depression	F32
Diabetes	E10, E11
Osteoporosis	M81
GORD	K21
Liver disease	K72 to K77
Lung cancer	C34
COPD	J44
Asthma	J45
Emphysema	J43
Bronchiectasis	J47
COVID 19	B97.29 U07.1
Pneumonia	J13 to J18 and J95.851
Obesity	E66.09, E66.1 E66.3, E66.8 E66.9 E66.2, E66.01
OSA	G47.3-G473.9
Invasive Mechanical ventilation	5A1935Z 5A1945Z, 5A1955Z,
Non Invasive Mechanical ventilation	5A09357, 5A09457, 5A09557

GORD: gastro-oesophageal reflux disease. COPD, chronic obstructive pulmonary disease;.OSA Obstructive Sleep Apnea

Table S2. Multivariable logistic regression to identify factors associated with severity in men and women hospitalized with a code for Alpha 1-Antitrypsin deficiency in Spain from 2016 to 2022.

		MEN	WOMEN	BOTH
		OR (95%CI)	OR (95%CI)	OR (95%CI)
Age groups, No. (%)	< 30 years	Reference	Reference	Reference
	30-49 years	1.02 (0.32-4.85	1.01(0.28-5.11)	1.01(0.45-3.54)
	50-69 years	1.25(0.47-3.39)	1.06(0.56-3.01)	1.12(0.71-2.55)
	>70 years	1.58(1.01-5.22)	1.85(1.04-6.28)	1.54(1.43-4.16)
Number of hospitalizations	One	Reference	Reference	Reference
	Two	1.25(0.72-2.65)	1.43(0.71-2.97)	1.32(0.92-1.94)
	Three or more	2.01(1.15-4.96)	2.04(1.08-5.23)	1.41(1.01-3.87)
Congestive Heart Failure	Yes	1.80(1.17-2.77)	1.71(1.02-2.98)	1.75(1.26-2.47)
Myocardial infarction	Yes	3.36(1.88-6.01)	-	2.57(1.57-4.22)
Liver disease	Yes	1.9(1.4-2.6)	1.61(1.02-2.65)	1.82(1.39-2.4)
Covid19	Yes	2.91(1.34-5.81)	-	2.55(-1.15-4.61)
Pneumonia	Yes	2.39(1.07-4.85)	-	2.03(1.01-3.97)
Sex	Men	NA	NA	1.39(1.10-1.75)

OR odds Ratio. CI Confidence interval. ICU Intensive care unit- NA Not available
